# 

**Published:** 1832-02

**Authors:** 


### MONTHLY LIST OF MEDICAL BOOKS.

[*Medical Works cannot be entered on this List unless a copy be sent for the purpose, the titles of Books having frequently been sent to us as published which have not been published for weeks, or even months, after.*]

Observations on the Nature of Malignant Cholera, with a view to establish correct Principles of its Prevention and Treatment: drawn up at the request of the Westminster Medical Society. By A. P. W. Philip, M.D., F.R.S. L. and E. —8vo. pp. 48. Renshaw and Rush, London.

On the Portable Sudatory, or Hot-air Bath; with Cases illustrative of its Medical Powers in various Disorders, and its great Utility in Cholera Morbus, with Directions for its Administration. Together with Remarks on the Applicability of Galvanism in the first Stage of that Disease. By M. La Beaume, Medical Galvanist, and Electrician in ordinary to the King, &c. Second Edition.—12mo. pp. 84. Highley, London.

Samouelle's Entomological Cabinet.—Renshaw and Rush, London.

*** This work is to be published in monthly parts, and to give a complete natural history of British Insects. From the contents of the first part, which is the only one yet published, we feel justified in predicting that the work will be instructive and amusing. The plates are neatly executed.

Published in Monthly Parts, THE CYCLOPÆDIA OF PRACTICAL MEDICINE; edited by JOHN FORBES, M.D., F.R.S., Physician to the Chichester Infirmary, &c.; ALEX. TWEEDIE, M.D., Physician to the London Fever Hospital, &c.; JOHN CONOLLY, M.D., late Professor of Medicine in the London University, &c. Part I. containing, Abdomen (Exploration of), by Dr. Forbes; Abortion, Dr. Lee; Abscess, Dr. Tweedie; Abstinence, Dr. M. Hall; Achor, Dr. Todd; Acne, Dr. Todd; Acupuncture, Dr. Elliotson; Age, Dr. Roget; Air (Change of), Dr. Clark; Alopecia, Dr. Todd; Alteratives, Dr. Conolly; Amaurosis, Dr. Jacob; Amenorrhœa, Dr. Locock; Anæmia, Dr. M. Hall; Anasarca, Dr. Darwall; Angina Pectoris, Dr. Forbes; Anodynes, Dr. Whiting; Anthelmintics, Dr. A. T. Thomson; Antiphlogistic Regimen, Dr. Barlow; Antispasmodics, Dr. Thomson; Aorta (Aneurism of), Dr. Hope.—Royal 8vo. pp. 112. Sherwin and Co., and Baldwin and Co., London.

A Series of Experiments performed for the purpose of shewing that Arteries may be Obliterated, without Ligature, Compression, or the Knife. By BENJ. PHILLIPS. 8vo. pp. 66. Longman and Co., London.

An Inquiry into the Medical Properties of Iodine, more particularly in Dropsy; also an Account of the Utility of Local Bloodletting in Hydrothorax and Bronchitis. Partially translated from the Latin of T. L. C. SCHROEDER VAN DER KOLK, Med. et Art. Obst. Doct. &c., by C. J. B. ALDIS, A.B.—8vo. pp. 32. Wilson, and Highley, London.

WEBER'S Anatomical Atlas, Parts III. and IV., with the English and German Explanations, containing Representations, of the full adult size, of, 1, First and Second Layers of Muscles of the Front of the Body; 2, Third and Fourth ditto; 3, First and Second Layers of Muscles of the Back of the Body; 4, Third and Fourth ditto; and Seven Supplementary Plates, with an Appendix to the Four Muscular Bodies, and Illustrations of the Anatomy of the Organs of Respiration and the Nose, of the Organs of Vision, of the Heart, of the Fœtal Circulation, and of the Membranes of the Ovum, of the Organs of Digestion.—Published by A. Schloss, foreign bookseller, and importer of anatomical models in wax; and may be had of all respectable booksellers.

*** We would recommend anatomical students to pay Mr. Schloss a visit, and to inspect the Anatomical Atlas of Weber: we are sure but few will hesitate to purchase it. It is by far the best and cheapest work of the kind that has fallen under our notice. The supplementary plates contained in the Appendix are also excellent, and very accurately represent various important parts of human anatomy.

Remarks on the Anatomy Bill now before Parliament, in a Letter to the Right Hon. the Lord Althorp, and given to the Members of either House, on their personal or written application to the Publisher. By G. J. GUTHRIE, F.R.S., Professor of Anatomy and Surgery to the Royal College of Surgeons; Surgeon to the Westminster Hospital, and to the Royal Westminster Ophthalmic Hospital, &c.—Sams, London.

Cholera, its Character and Treatment; with Remarks on the Identity of the Indian and English, and a particular Reference to the Disease as now existent at Newcastle and its Neighbourhood. By C. T. THACKRAH.—8vo. pp. 60. Baines, Leeds; Longman and Co., London.

Letters on the Cholera, by WHITELAW AINSLIE, M.D., M.R.A.S., M.R.S.E.—Wilson, London.

Observations on the Medical Treatment of Insanity. By EDW. J. SEYMOUR, M.D., Physician to St. George's Hospital, Consulting Physician to the Seaman's Hospital, and Physician to his Royal Highness the Duke of Sussex.—8vo. pp. 95. Longman, London.

A History of British Animals; exhibiting the Descriptive Characters and Systematical Arrangement of the Genera and Species of Quadrupeds, Birds, Reptiles, Fishes, Mollusca, and Radiata of the United Kingdom; including the Indigenous, Extirpated, and Extinct Kinds, together with periodical and occasional Visitants. By JOHN FLEMING, D.D., F.R.S.E., M.W.S., &c., Minister of Flisk, Fifeshire, and author of the "Philosophy of Zoology."—8vo. pp. 565. Bell and Bradfute, Edinburgh, and James Duncan, London.

Part III. The Principles and Practice of Obstetric Medicine, in a Series of Systematic Dissertations on Midwifery, and on the Diseases of Women and Children: illustrated by numerous Plates. By D. D. DAVIS, M.D., M.R.S.L.; Professor of Midwifery in the University of London, &c.—John Taylor, London.

### NOTICES.

*Communications have been received from* MR. ADAMS, MR. G. BENNETT, MR. CÆSAR HAWKINS, *&c.*

